# Dietary glucoraphanin prevents the onset of psychosis in the adult offspring after maternal immune activation

**DOI:** 10.1038/s41598-018-20538-3

**Published:** 2018-02-01

**Authors:** Akiko Matsuura, Tamaki Ishima, Yuko Fujita, Yoshimi Iwayama, Shunsuke Hasegawa, Ryouka Kawahara-Miki, Motoko Maekawa, Manabu Toyoshima, Yusuke Ushida, Hiroyuki Suganuma, Satoshi Kida, Takeo Yoshikawa, Masaomi Iyo, Kenji Hashimoto

**Affiliations:** 1 Division of Clinical Neuroscience, Chiba University Center for Forensic Mental Health, Chiba, 260–8670 Japan; 2 Department of Psychiatry, Graduate School of Medicine, Chiba University, Chiba, 260–8670 Japan; 3 Laboratory for Molecular Psychiatry, RIKEN Brain Science Institute, Saitama, 351–0198 Japan; 4 Department of Bioscience, Faculty of Applied Bioscience, Tokyo University of Agriculture, Tokyo, 156–8502 Japan; 5 NODAI Genome Research Center, Tokyo University of Agriculture, Tokyo, 156–8502 Japan; 6 Innovation Division, Kagome Co., Ltd., Tochigi, 329–2762 Japan

## Abstract

Maternal immune activation (MIA) contributes to behavioral abnormalities relevant to schizophrenia in adult offspring, although the molecular mechanisms underlying MIA-induced behavioral changes remain unclear. Here we demonstrated that dietary intake of glucoraphanin (GF), the precursor of a natural antioxidant sulforaphane, during juvenile and adolescent stages prevented cognitive deficits and loss of parvalbumin (PV) immunoreactivity in the medial prefrontal cortex (mPFC) of adult offspring after MIA. Gene set enrichment analysis by RNA sequencing showed that MIA caused abnormal expression of centrosome-related genes in the PFC and hippocampus of adult offspring, and that dietary intake of GF improved these abnormal gene expressions. Particularly, MIA increased the expression of suppressor of fermentation-induced loss of stress resistance protein 1 (*Sfi1*) mRNA in the PFC and hippocampus of adult offspring, and dietary intake of GF prevented the expression of *Sfi1* mRNA in these regions. Interestingly, we found altered expression of SFI1 in the postmortem brains and *SFI1* mRNA in hair follicle cells from patients with schizophrenia compared with controls. Overall, these data suggest that centrosome-related genes may play a role in the onset of psychosis in offspring after MIA. Therefore, dietary intake of GF-rich vegetables in high-risk psychosis subjects may prevent the transition to psychosis in young adulthood.

## Introduction

The onset of schizophrenia, a chronic and severe mental disorder, usually occurs in young adulthood^1,2^. Among three major symptoms (e.g. positive symptom, negative symptom, cognitive impairment), cognitive impairment is the core symptom of schizophrenia^3–5^. Interestingly, cognitive impairment can be detected before the onset of schizophrenia^6,7^. Therefore, there are increasing interests in the potential benefit of early intervention by nutritional antioxidants in schizophrenia since oxidative stress plays a role in the prodromal symptoms of schizophrenia^8–14^.

**Figure 1:**
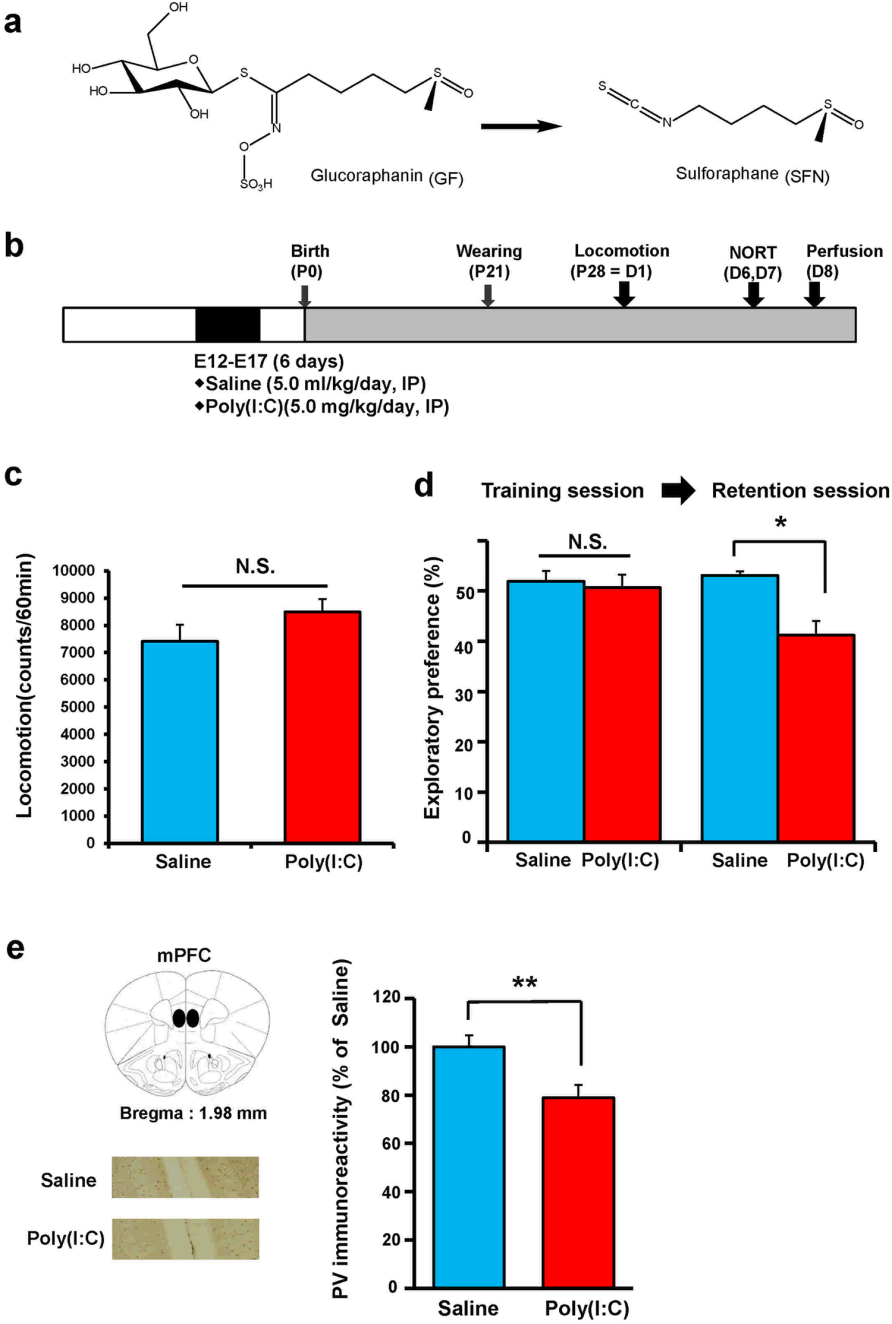
Cognitive deficits in the juvenile offspring after prenatal poly(I:C) exposure. (**a**) Chemical structure of sulphoraphane (SFN) and its precursor glucoraphanin (GF). (**b**) Schedule of treatment and behavioral tests. Saline (5.0 ml/kg/day) or poly(I:C) (5.0 mg/kg/day from E12 to E17) was injected into pregnant mice. Behavioral tests including locomotion (LMT: P28 = D1) and novel object recognition test (NORT: D6 and D7) were performed. Brain samples for immunohistochemistry were collected at D8. (**c**) Locomotion: There was no difference (P = 0.908) between ploy(I:C) group and saline-treated group. The value is expressed as the mean ± S.E.M. (n = 8). (**d**) NORT: There was no difference (P = 0.282) between the two groups in the training session. In the retention session, the exploratory preference of poly(I:C) group was significantly (P = 0.048) lower than saline-treated group. *P < 0.01 compared with saline-treated group. The value is expressed as the mean ± S.E.M. (n = 8). (**e**) Brain atlas of medial prefrontal cortex (mPFC) and representative data of PV-immunoreactivity in the mPFC of juvenile offspring after MIA: The PV-immunoreactivity in the mPFC of poly(I:C)-treated group was significantly (P = 0.005) lower than that of saline-treated group. **P < 0.01, compared with saline-treated group. The value is expressed as the mean ± S.E.M. (n = 8).

The transcription factor Nuclear factor (erythroid 2-derived)-like 2 (Nrf2) and Keap1 (Kelch-like erythroid cell-derived protein with CNC homology [ECH]-associated protein 1) system plays a central role in cellular defense against oxidative stress^15,16^. Thus, the Keap1–Nrf2 system is involved in attenuating oxidative stress-associated pathogenesis of a number of disorders^15,16^. The potent antioxidant and naturally occurring compound sulforaphane (SFN; 1-isothiocyanato-4-methylsulfinylbutane) is an organosulfur compound derived from a glucosinolate precursor glucoraphanin (GF) (Fig. 1a), which is found in cruciferous vegetables such as broccoli^17,18^. We reported that SFN could attenuate behavioral abnormalities in mice after the administration of a psychostimulant methamphetamine^19^ or *N*-methyl-D-aspartate receptor (NMDAR) antagonist phencyclidine (PCP)^20^. Furthermore, we reported that dietary intake of 0.1% GF food pellet during juvenile and adolescent stages could prevent the onset of cognitive deficits after repeated PCP administration^21^. These findings suggest that SFN (or GF) may have prophylactic and therapeutic effects on cognitive impairment and psychosis in psychiatric disorders such as schizophrenia.

Emerging epidemiologic data state that gestational exposure to infection contributes to the etiology of schizophrenia^22–24^. Maternal immune activation (MIA) using polyriboinosinic-polyribocytidilic acid [(poly(I:C)], a Toll-like receptor 3 agonist, has been widely used as a neurodevelopmental animal model for schizophrenia^25–32^. MIA contributes to behavioral abnormalities relevant to schizophrenia in adult offspring, although the molecular mechanisms underlying MIA-induced behavioral changes remain unclear^26,27^.

The present study aimed to examine whether dietary intake of GF food pellets during juvenile and adolescent stages could prevent the onset of behavioral abnormalities relevant to schizophrenia in adult offspring after MIA. Furthermore, we examined the molecular mechanisms of GF’s beneficial effects in MIA model using RNA-sequencing and gene set enrichment analysis (GSEA).

## Results

### Behavioral data and PV-immunohistochemistry of juvenile offspring after MIA

First, we performed behavioral tests (locomotion and NORT) and parvalbumin (PV) immunohistochemistry in juvenile offspring after MIA because the reduction of PV immunoreactivity in the medial prefrontal cortex (mPFC) might be related with cognitive impairment and psychosis^28,33,34^. Behavioral tests of juvenile offspring were performed at P28–P35 after prenatal poly(I:C) (5.0 mg/kg/day from E12 to E17) injections (Fig. 1b). In the open field test, there was no difference between the control and poly(I:C)-treated groups (Fig. 1c). In NORT, there was no difference between the two groups during the training session (Fig. 1d). However, during the retention session, the exploratory preference of the poly(I:C)-treated group was significantly lower than that of the control group (Fig. 1d). PV-immunoreactivity (PV-IR) in the mPFC of juvenile offspring of the poly(I:C)-treated group was significantly lower than that in the saline-treated (control) group (Fig. 1e). These findings suggest that juvenile offspring of prenatal poly(I:C)-treated mice showed the reduction of PV-IR in the mPFC, resulting in cognitive deficits.

### Gene expression of Keap1 and Nrf2 in the prefrontal cortex and hippocampus from juvenile offspring after MIA

**Figure 2:**
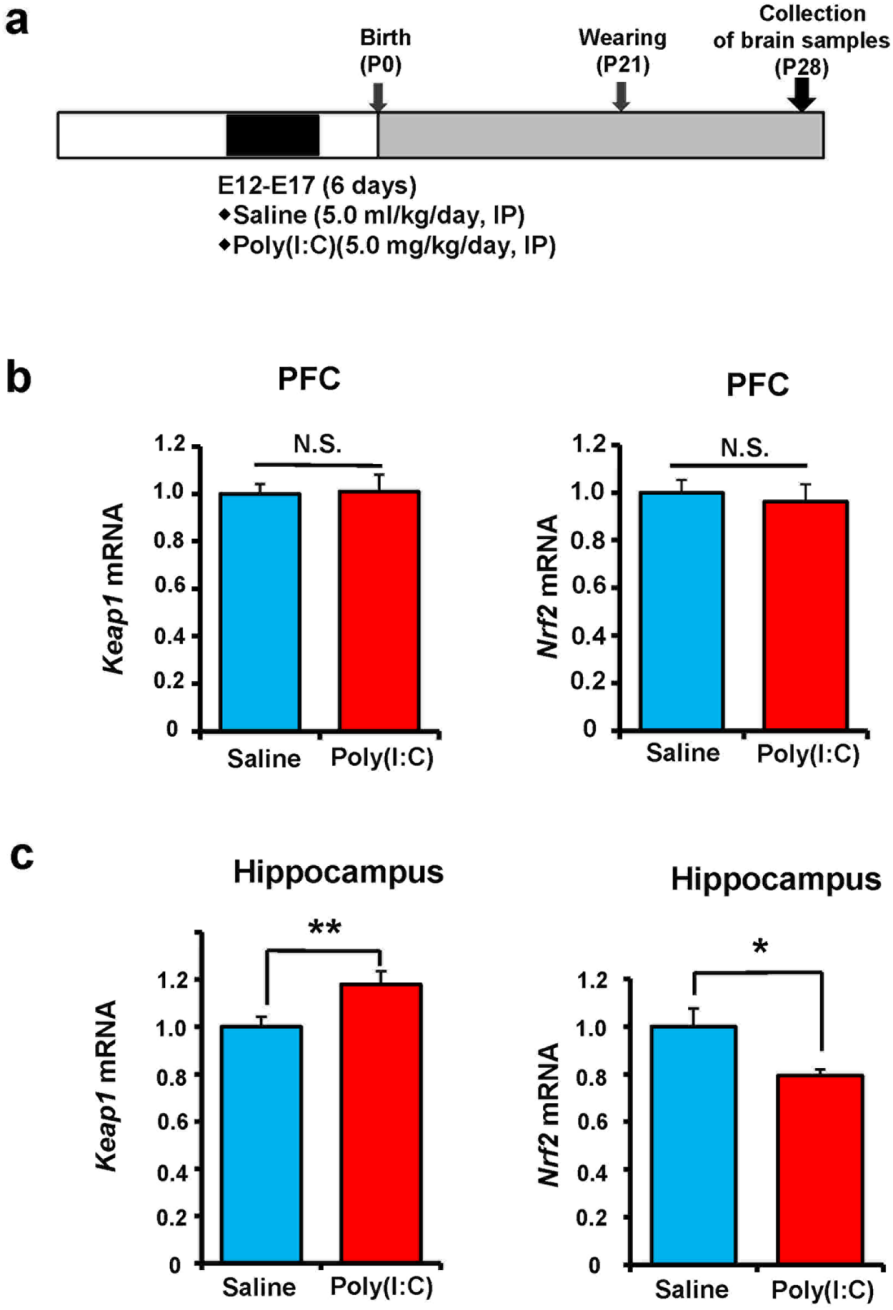
Gene expression of *Keap1* and *Nrf2* in the PFC and hippocampus of juvenile offspring after prenatal poly(I:C) exposure. (**a**) Schedule of treatment and collection of brain samples. (**b**) There were no changes for gene expression of *Keap1* (P = 0.903) and *Nrf2* (P = 0.794) in the PFC. (**c**) There were significant changes for gene expression of *Keap1* (P = 0.012) and *Nrf2* (P = 0.023) in the hippocampus. Data represent the mean ± S.E.M. (n = 10 for saline-treated group, n = 14 for poly(I:C) group). *P < 0.05, **P < 0.01 compared with saline-treated group. NS: not significant.

We measured *Keap1* and *Nrf2* gene expressions in the PFC and hippocampus of juvenile offspring (4-week-old) after MIA (Fig. 2a). The expressions of these two genes in the PFC were not different between the two groups (Fig. 2b). However, *Keap1* mRNA expression in the hippocampus was significantly higher, whereas *Nrf2* mRNA expression in the poly(I:C)-treated group was significantly lower than that of control group (Fig. 2c). These findings suggest that abnormalities in Keap1–Nrf2 signaling in the hippocampus may play a role in the behavioral abnormality of juvenile offspring after prenatal poly(I:C) injections.

### Effects of dietary intake of 0.1% GF food pellets during juvenile and adolescent stages on cognitive deficits and reduction of PV-IR in the mPFC of adult offspring after MIA

**Figure 3:**
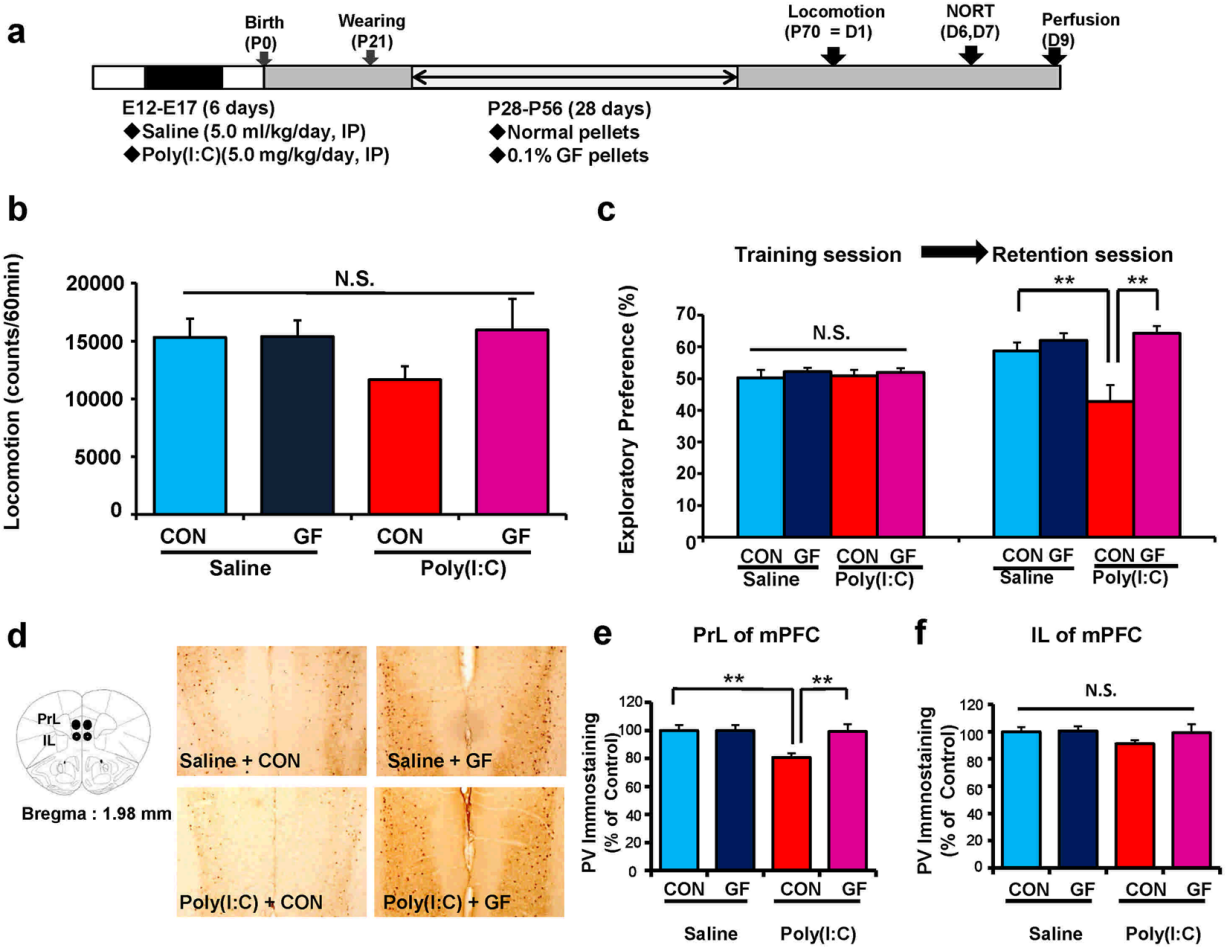
Effects of dietary intake of 0.1% GF on cognitive deficits in the adult offspring after prenatal poly(I:C) exposure. (**a)** Schedule of treatment and behavioral tests. Saline (5 ml/kg/day) or poly(I:C) (5.0 mg/kg/day from E12 to E17) was injected into pregnant mice. Normal food pellets or 0.1% GF food pellets were given to juvenile offspring from D28 to D56. Subsequently, normal food pellets were given to all mice for 14 days (D57-). Brain samples were collected at D70. (**b)** Locomotion: There was no difference (poly(I:C): F_1,29_ = 0.7555, P = 0.3919; GF: F_1,29_ = 1.515, P = 0.2282; interaction: F_1,29_ = 1.434, P = 0.2408) among the four groups. The value is expressed as the mean ± S.E.M. (n = 8 for saline + control pellet group, n = 8 for saline + GF pellet group, n = 9 for poly(I:C) + control pellet group, n = 8 for poly(I:C) + GF pellet group). (**c)** NORT: There was no difference (poly(I:C): F_1,29_ = 0.019, P = 0.891; GF: F_1,29_ = 0.891, P = 0.390; interaction: F_1,29_ = 0.057, P = 0.812) among the four groups in the training session. In the retention session, the exploratory preference of poly(I:C) + GF pellet group was significantly (poly(I:C): F_1,29_ = 3.346, P = 0.048; GF: F_1,29_ = 10.943, P = 0.003; interaction: F_1,29_ = 5.832, P = 0.022) higher than poly(I:C) + control pellet group. **P < 0.01 compared with poly(I:C) + control pellet group. The value is expressed as the mean ± S.E.M. (n = 8 for saline + control pellet group, n = 8 for saline + GF pellet group, n = 9 for poly(I:C) + control pellet group, n = 8 for poly(I:C) + GF pellet group). (**d)** Brain atlas of PrL and IL regions of mPFC and representative data of PV-immunoreactivity in the mPFC of juvenile offspring. (**e**) The PV-immunoreactivity in the PrL of mPFC of poly(I:C) + GF pellet group was significantly (poly(I:C): F_1,29_ = 5.798, P = 0.023; GF: F_1,29_ = 4.992, P = 0.033; interaction: F_1,29_ = 4.992, P = 0.033) higher than that of poly(I:C) + control pellet group. **P < 0.01, compared with poly(I:C) + control pellet group. The value is expressed as the mean ± S.E.M. (n = 8). (**f**) The PV-immunoreactivity in the IL of mPFC was not different (poly(I:C): F_1,29_ = 1.597, P = 0.216; GF: F_1,29_ = 1.113, P = 0.300; interaction: F_1,29_ = 0.8391, P = 0.367) among the four groups. The value is expressed as the mean ± S.E.M. (n = 8).

We examined whether dietary intake of 0.1% GF food pellets during juvenile and adolescent stages could prevent cognitive deficits and reduction of PV-IR in the mPFC of adult offspring after MIA. Offspring (4-week-old) were divided into normal food pellet and 0.1% GF-containing food pellet groups. The mice were given free access to water and both the food pellets that were specifically designed for mice for 4 weeks (P28–P56). Subsequently, normal food pellets were given to both the groups. Behavioral tests of adult offspring were performed during adulthood (P70–P84) after prenatal poly(I:C) injections (Fig. 3a). In the open field test, locomotion was unchanged among the four groups (Fig. 3b). There was no difference among the four groups in NORT training sessions. However, in the retention session of NORT, the exploratory preference of the poly(I:C) + GF group was significantly higher than that of the poly(I:C) + control group (Fig. 3c). These findings suggest that dietary intake of 0.1% GF pellets from P28 to P56 improved cognitive deficits in adult offspring after MIA.

Furthermore, we performed PV immunohistochemistry at adulthood (10 weeks) (Fig. 3d and e). PV-IR in the PrL (not IL) of the mPFC of the poly(I:C) + GF group was significantly higher than that in the poly(I:C) + control group (Fig. 3e). These findings suggest that dietary intake of 0.1% GF food pellets from P28 to P56 prevented the reduction of PV-IR in the PrL of the mPFC in adult offspring after MIA.

### RNA-sequencing and GSEA

**Figure 4:**
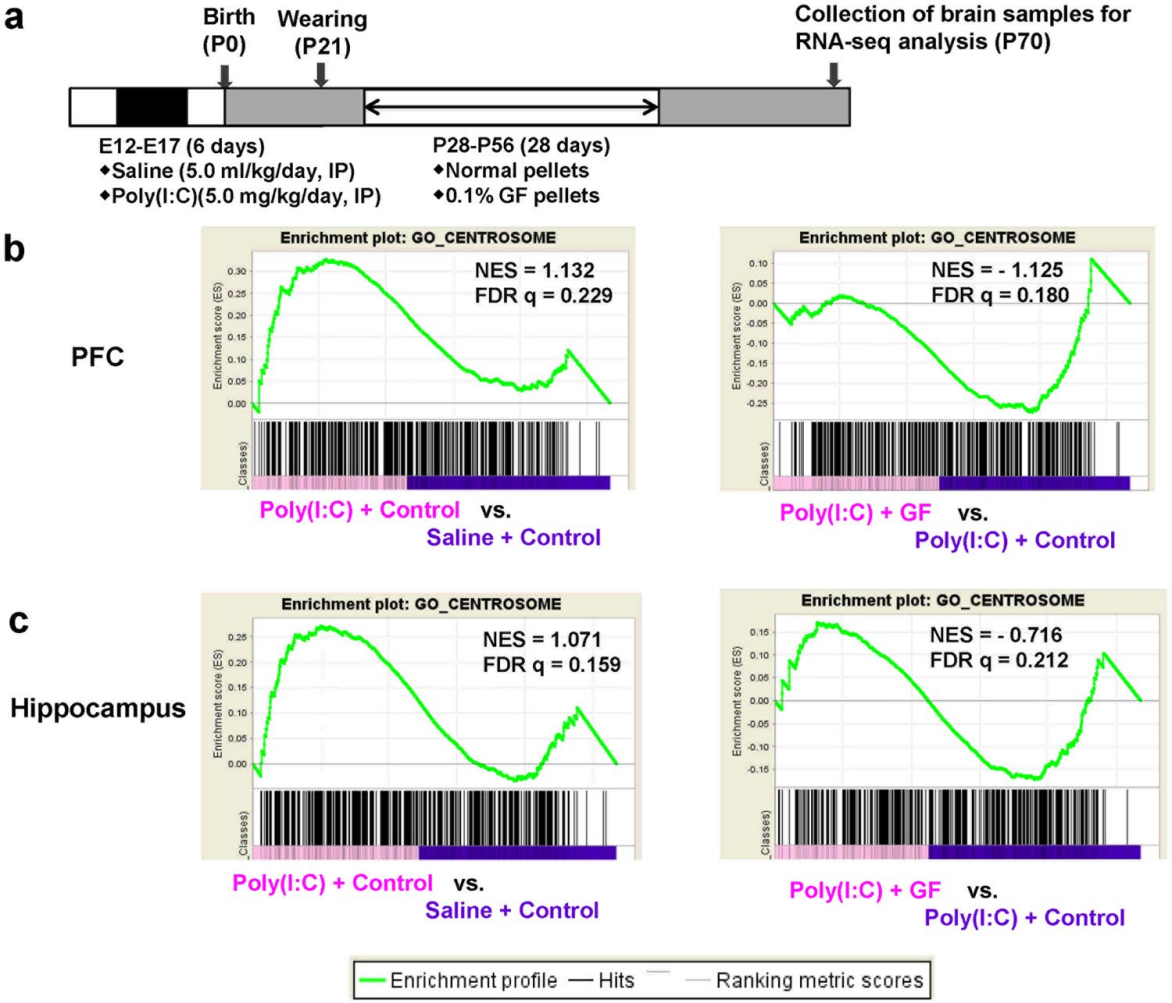
RNA-sequencing analysis and GSEA of brain samples from adult offspring after prenatal poly(I:C) exposure. (**a)** Schedule of treatment and behavioral tests. Saline (5 ml/kg/day) or poly(I:C) (5.0 mg/kg/day from E12 to E17) was injected into pregnant mice. Normal food pellets or 0.1% GF food pellets were given to juvenile offspring from D28 to D56. Subsequently, normal food pellets were given to all mice for 14 days (D57-). Brain samples were collected at D70, and RNA-sequencing analysis was performed. (**b**,**c**) The GSEA (gene set enrichment analysis) plots showing enrichment of centrosome-related gene sets in the PFC (**b**) and Hippocampus (**c**). The normalized enrichment scores (NES) and false discovery rate q value (FDR q) were indicated.

**Figure 5:**
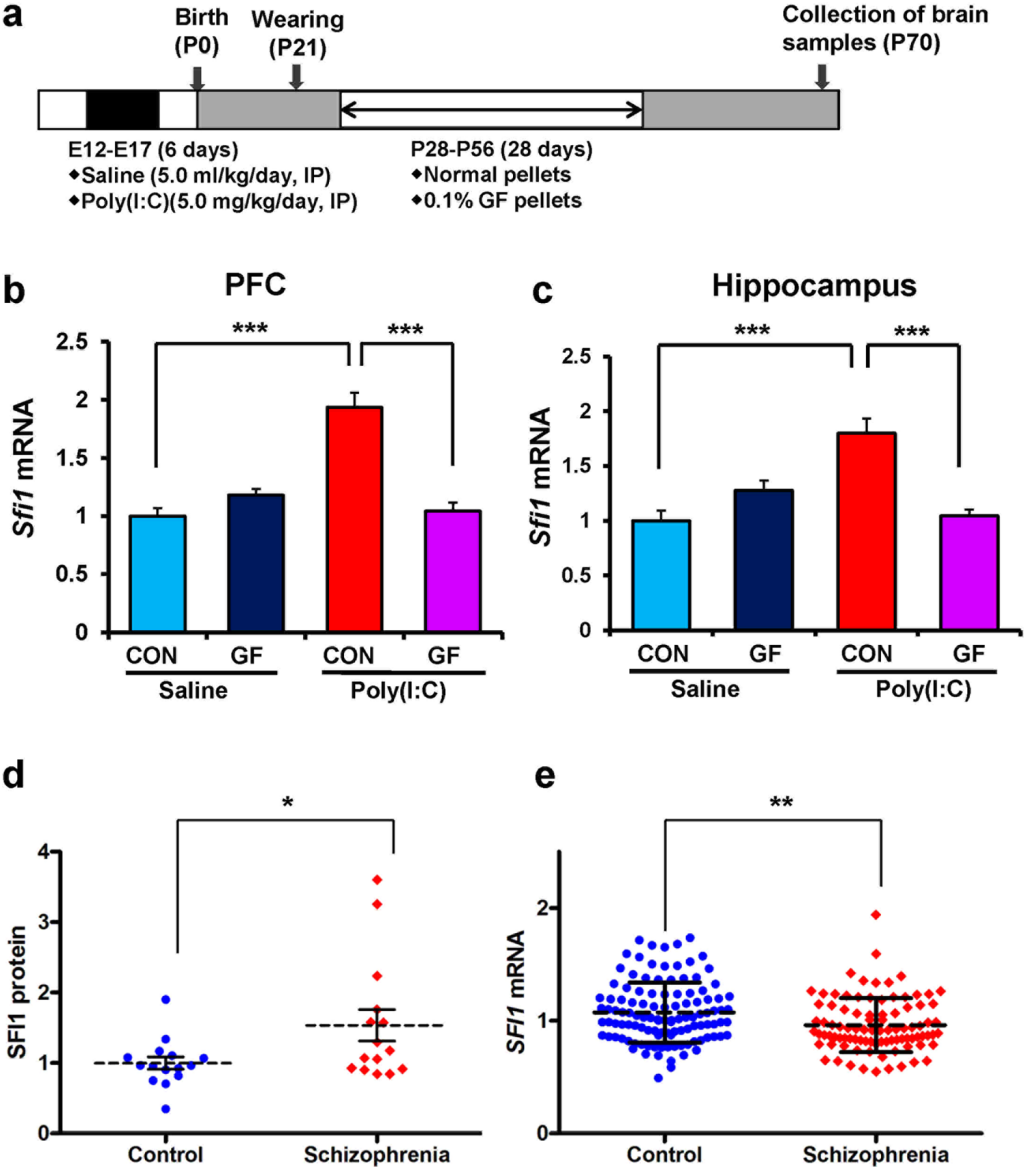
Expression of *Sfi1* mRNA in the PFC and hippocampus from the adult offspring after prenatal poly(I:C) exposure and expression of SFI1 protein and *SFI1* mRNA in schizophrenia. (**a**) Schedule of treatment and behavioral tests. Saline (5 ml/kg/day) or poly(I:C) (5.0 mg/kg/day from E12 to E17) was injected into pregnant mice. Normal food pellets or 0.1% GF food pellets were given to juvenile offspring from D28 to D56. Subsequently, normal food pellets were given to all mice for 14 days (D57-). Brain samples were collected at D70. (**b**): *Sfi1* mRNA in the PFC: There was significant effects (poly(I:C): F_1,22_ = 25.39, P < 0.001; GF: F_1,22_ = 20.28, P < 0.001; interaction: F_1,22_ = 45.97, P < 0.001). (**c**) *Sfi1* mRNA in the hippocampus: There was significant effects (poly(I:C): F_1,22_ = 9.29, P = 0.006; GF: F_1,22_ = 6.65, P = 0.017; interaction: F_1,22_ = 31.01, P < 0.001). The value is expressed as the mean ± S.E.M. (n = 5–7). ***P < 0.001 compared to poly(I:C) + control group. (**d**) Expression of SFI1 protein in the parietal cortex from patients (n = 15) with schizophrenia was significantly (P = 0.034) higher than that of controls (n = 15). The value is expressed as the mean ± S.D. *P < 0.05 compared to control group. (**e**) Expression of *SFI1* mRNA in the hair-follicle from patients (n = 94) with schizophrenia was significantly (P = 0.002) lower than that of controls (n = 117). The value is expressed as the mean ± S.D. **P < 0.01 compared to control group.

To study the molecular targets for the beneficial effects of dietary intake of 0.1% GF food pellets, we performed RNA sequencing of the PFC and hippocampus from the four groups of adult offspring. The most important finding using gene set enrichment analysis was that centrosome-related genes played a key role in the molecular mechanisms underlying the beneficial effects of GF dietary intake in the MIA model (Fig. 4a–c). The centrosome is involved in many different cell processes, particularly cell division, cell migration, and differentiation. Accumulating evidence suggests a central role of centrosome at late stages of neuronal development^35^. Among the centrosome genes, we found a significant change in suppressor of fermentation-induced loss of stress resistance 1 (*Sfi1*) expression, which is localized in the centriole and involved in cell division^36,37^. We measured the expression of *Sfi1* mRNA and SFI1 levels in subsequent experiments. The expression of *Sfi1* mRNA in the PFC and hippocampus of adult offspring was significantly increased by prenatal poly(I:C) injection, whereas dietary intake of 0.1% GF food pellets significantly blocked the increase in this gene expression (Figs. 5a–c).

### Measurements of SFI1 protein in the postmortem brain samples and SFI1 mRNA in hair follicle cells from schizophrenia

Western blot analysis showed that SFI1 expression in the parietal cortex of patients with schizophrenia (Table 1) was significantly higher than that of controls (Fig. 5d). To test whether the expression of *SFI1* mRNA is associated with the pathophysiology of schizophrenia, we measured *SFI1* mRNA expression in hair follicle cells from schizophrenic and healthy control subjects (Table 2)^33,34^. The *SFI1* mRNA expression in the hair follicle cells of patients with schizophrenia was significantly lower than that of healthy controls (Fig. 5e). Correlation analyses demonstrated no significant effects of age, dose of antipsychotics, or duration of illness on *SFI1* mRNA expression.

**Table 1:** Characteristics of the postmortem samples from Neuropathology Consortium of the Stanley Medical Research Institute.

Characteristics	Control (n = 15)	Schizophrenia (n = 15)	P value
Age at death (years)	48.1 ± 10.7 (29–68)	44.5 ± 13.1 (25–62)	0.425^a^
Gender (male/female)	9/6	9/6	1.000^b^
PMI (hrs)	23.7 ± 9.95	33.7 ± 14.6	0.038^a^
Brain pH	6.27 ± 0.24	6.16 ± 0.26	0.250^a^
Brain hemispheres (right/left)	7/8	6/9	0.713^b^
Brain weight (g)	1501.0 ± 164.1	1471.7 ± 108.2	0.568^a^
Storage days	338.2 ± 234.2	621.1 ± 233.1	0.003^a^
Age of onset (years)		23.2 ± 8.0	
Duration of disease (years)		21.3 ± 11.4	
Fluphenazine equivalent (mg)		52267 ± 62062 (1 never)	

The data are shown the mean ± S.D. PMI: postmortem interval. ^a^Unpaired t-test, ^b^*x*^2^ test for independence.

**Table 2:** Characteristics of the hair-follicle samples from control and schizophrenia.

Characteristics	Control (n = 117)	Schizophrenia (n = 94)	P value
Age (years)	43.9 ± 13.1 (21–73)	50.5 ± 11.8 (21–72)	<0.001^a^
Gender (male/female)	50/67	49/45	0.174^b^

See the ref.^24^. The data are shown the mean ± S.D. ^a^Unpaired t-test, ^b^*x*^2^ test for independence.

## Discussion

In the present study, we found that dietary intake of 0.1% GF food pellets during the juvenile and adolescent stages of offspring after MIA prevented cognitive deficits and reduction of PV-IR in the PrL of the mPFC at adulthood after MIA. Furthermore, centrosome signaling via Sfi1 might play a role in the development of behavioral abnormalities in adult offspring after MIA, as well as in the beneficial effects of 0.1% GF dietary intake. Finally, we found altered expressions of SFI1 in the parietal cortex and *SFI1* mRNA in hair follicle cells of patients with schizophrenia, suggesting that abnormal expression of SFI1 plays a role in the pathogenesis of schizophrenia. These all findings suggest that altered centrosome signaling through Sfi1 might play a role in the development of behavioral abnormalities after MIA as well as in the beneficial effects of GF dietary intake. Therefore, it is likely that supplementation with GF-rich food in subjects with a high-risk for psychosis has prophylactic effects on the psychotic behaviors relevant to schizophrenia and related disorders in adulthood.

We found cognitive deficits of juvenile offspring from poly(I:C)-treated mice, consistent with previous reports^28,29^. Since cognitive impairment is seen in adolescent and young adult with a high risk for psychosis^6,7^, it seems that the juvenile offspring after MIA may be at the prodromal stage for psychosis^28,29^. Furthermore, we found reduction of PV-IR in the mPFC at juvenile offspring after MIA, which is consistent with the previous study^28^. It is well known that reduction of PV-IR in the PFC may contribute to the pathophysiology of schizophrenia^33,34^. Therefore, it is likely that reduction of PV-IR in the mPFC may play a crucial role in the cognitive deficits of juvenile offspring after MIA.

Furthermore, we found abnormal expression of *Keap1 and Nrf2* genes in the hippocampus of juvenile offspring after prenatal poly(I:C) exposure, although expression of these genes in the PFC remained the same. Thus, it seems that abnormalities in the Keap1-Nrf2 system in the hippocampus might play a role in the cognitive deficits seen in juvenile offspring after MIA. Recently, we reported decreased gene expression of *N*-methyl-D-aspartate (NMDA) receptor subtypes, *Grin1*, *Grin2a*, and *Grin2b* in the hippocampus (but not PFC) of juvenile offspring after prenatal poly(I:C) exposure^29^. Taken together, it is likely that MIA can interfere with Keap1-Nrf2 system and NMDA receptor in the hippocampus during brain development, resulting in cognitive deficits in juvenile and adult offspring. A recent study showed that the expressions of Keap1 and Nrf2 in the parietal cortex from patients with schizophrenia were lower than those of controls, suggesting that Keap-Nrf2 signaling may play a role in the pathophysiology of schizophrenia^38^. Nonetheless, further detailed studies on how prenatal poly(I:C) exposure induces the Keap1-Nrf2 system and behavioral abnormalities in juvenile and adulthood are needed.

We found reduction of PV-IR in the PrL, but not IL, of mPFC at adult offspring after MIA, consistent with the previous findings^28,39^. Reduction of PV-IR cells in the PFC is suggested to contribute to the pathophysiology of schizophrenia^33,34^. Interestingly, we found that dietary intake of 0.1% GF food pellet at 4–8 weeks of age (similar to juvenile and adolescent stages in human) in poly(I:C) offspring could treat or prevent cognitive deficits and reduction of PV-IR at adulthood after MIA. Prenatal infection may contribute to the onset of neurodevelopmental disorders in their offspring^40^, and alterations in the Keap1-Nrf2 signaling in the hippocampus may play a role in MIA-induced abnormal brain neurodevelopment. Previously, we reported that dietary intake of 0.1% GF food pellet might prevent the onset of cognitive deficits and reduction of PV-IR in the mPFC after repeated PCP administration^21^. Furthermore, we also reported that dietary intake of 0.1% GF food pellet could prevent the onset of depression-like phenotype in mice after chronic social defeat stress^41^ or inflammation^42^. Taken together, it is likely that early intervention with GF (or SFN)-rich foods during juvenile and adolescent stages might have prophylactic and therapeutic effects on abnormal behaviors in psychiatric disorders, such as schizophrenia and depression^21,41,42^.

The centrosome is the main microtubule organizer in mammalian cells, participating in a variety of processes from cell polarization to cell division^35^. In this study, *SFI1* mRNA expression in the hair follicle cells of patients with schizophrenia was lower than that of healthy controls although SFI1 protein expression in the parietal cortex from patients with schizophrenia was higher than that of controls. The reasons underlying this discrepancy are currently unclear, the findings are similar to the previous report (*FABP4* mRNA in hair follicle and postmortem brain)^43^. Mutations in genes encoding centrosomal components are associated with several neurological and psychiatric disorders, such as schizophrenia. For example, mutations in the centrosomal genes lissencephaly 1 (*LIS1*) and doublecortin (*DCX*), have been linked to neurodevelopmental disorders^35^, while the centrosome gene disrupted-in-schizophrenia-1 *(DISC1*) is a candidate susceptibility gene for schizophrenia^44^. A recent study demonstrated that DISC1 regulates Ndel1’s kinetochore attachment, but not its centrosome localization, during mitosis, and that disrupting DISC1/Ndel1 complex formation prolongs mitotic length and interferes with cell cycle progression in human cells^45^. Furthermore, alterations in the expression of *Sfi1* in the striatum and hippocampus of 39,X^Y*^O mice, a genetic model of neurodevelopmental disorder have been reported^46^. Moreover, copy number variants encompassing *SFI1* on chromosome 22q12.2 have been identified in neurodevelopmental disorders, such as autism spectrum disorder (ASD)^47–49^. It is noteworthy that beneficial effects of SFN have been shown in young subjects with ASD^50^.

Adolescence is more vulnerable to psychiatric disorders since adolescence is a critical period of brain neurodevelopment^1,51^. The study also supports for the deleterious effects of early brain insult on abnormalities in brain neurodevelopment^21,28,52^. Furthermore, it is known that adolescence is the peak time for the onset of a number of psychiatric disorders. Therefore, it seems that cognitive impairment at adolescence may present prodromal symptoms for later onset of these disorders^28–30^. Therefore, it is likely that early intervention by the nutritional antioxidants in young peoples at high risk for psychosis might play a crucial role in preventing the onset of psychosis.

This paper has some limitations: Postmortem interval (PMI) and storage days of postmortem brain samples were significantly different from the two groups. Furthermore, age from the hair-follicle samples was significantly different from the two groups. These parameters may affect the expression of SFI1 protein and *SFI1* mRNA in the human samples. Further study using controls with matched for these parameters will be needed.

In conclusion, the present data suggest that abnormal modulation of centrosome-related genes plays a role in the behavioral abnormality and reduction of PV-IR in the mPFC of offspring after MIA. Interesting, through the centrosome modulation, dietary intake of 0.1% GF during juvenile and adolescent stage could prevent the onset of psychosis in the adult offspring after MIA. Finally, supplementation of SFN (or GF)-rich vegetables in young peoples at high risk for psychosis may prevent the transition to actual psychosis.

## Materials and Methods

### Animals

Pregnant ddY mice (embryo at the 5^th^ day (E5), 9–10 weeks old) were purchased from Japan SLC Inc. (Hamamatsu, Shizuoka, Japan). Pregnant mice in each clear polycarbonate cage (22.5 × 33.8 × 14.0 cm) one by one were under a controlled 12/12 h light–dark cycle (lights on from 07:00 a.m. to 07:00 p.m.), with room temperature at 23 ± 1 °C and humidity at 55 ± 5%. The mice were kept free access to water and pellets. The experimental procedure using animals was approved by the Chiba University Laboratory Animal Care and Use Committee. This study was carried out in strict accordance with the recommendations in the Guide for the Care and Use of Laboratory Animals of the National Institutes of Health, USA.

The study using postmortem brain samples was approved by Research Ethics Committee of the Graduate School of Medicine, Chiba University. The study using the samples of scalp hair-follicle was also approved by the Ethics Committees of RIKEN and all participating institutes^43,53^. This study was conducted according to the principles expressed in the Declaration of Helsinki. All subjects gave written, informed consent to participate in the study after the study protocols and objectives were explained^43,53^.

### Administration of poly(I:C) into pregnant mice

The schedule of poly(I:C) treatment was performed as reported previously^25,28–30^. The pregnant mice were injected intraperitoneally (i.p.) for six consecutive days from E12 to E17 with poly(I:C) (5.0 mg/kg/day, Sigma-Aldrich Co. Ltd., St. Louis, MO, USA) dissolved in physiological saline, or an equivalent volume (5 ml/kg) of saline. The male offspring were separated from their mothers after 3 weeks, and mice were caged each three or five in the groups.

### Preparation of 0.1% glucoraphanin (GF) food pellets

Food pellets (CE-2; Japan CLEA, Ltd., Tokyo, Japan) containing 0.1% glucoraphanin (GF) were prepared as reported previously^20,41,42^. Basically, broccoli sprout extract powder containing GF (a precursor of SFN) was industrially produced by KAGOME CO., LTD (Nasushiobara, Tochigi, Japan). In brief, broccoli sprout was grown from specially selected seeds (Brassica Protection Products LLC., Baltimore, MD, USA) for 1 day after the germination. On the 1st day broccoli sprout was plunged into boiling water and maintained at 95 °C for 30 minutes, and the sprout residues was removed by filtration. The boiling water extract was mixed with a waxy corn starch dextrin and then spray dried to yield the broccoli sprout extract powder containing 135 mg (approx. 0.31 mmol) of GF per gram. For preparing the animal diet containing 0.1% GF (approx. 2.3 mmol GF per 1 kg-diet), the extract powder was mixed with a basal diet CE-2, and then pelletized at a processing facility (Oriental Yeast Co., ltd., Tokyo, Japan). The GF content in the diet was determined by high performance liquid chromatography as previously described^21,41,42^.

### Behavioral analysis

Locomotion and the novel object recognition test (NORT) were performed as reported previously^20,28,29^. Locomotor Activity**:** Both horizontal and rearing activity were monitored by an infrared ray passive sensor system (SCANET-SV10, Melquest Ltd., Toyama, Japan), and activity was integrated every minute. Individual mice were placed in activity chambers and allowed 1 hour of free exploration as spontaneous activity.

Novel Object Recognition Test (NORT): Mice were habituated for 10 minutes in the box for 3 straight days. At 4th day, two objects (differing in shape and color but of similar size) were placed in the box 35.5 cm apart (symmetrically), and each animal was allowed to explore in the box for 5 minutes. The animals were considered to be exploring the object when the head of the animal was both facing and within 2.54 cm of the object or when any part of the body, except for the tail was touching the object. The time that mice spent exploring each object was recorded. After training, mice were immediately returned to their home cages, and the box and objects were cleaned with 75% ethanol, to avoid any possible instinctive odorant cues. Retention tests were carried out at one-day intervals, following the respective training. During the retention test, each mouse was reintroduced into their original test box, and one of the training objects was replaced by a novel object. The mice were then allowed to explore freely for 5 minutes, and the time spent exploring each object was recorded. Throughout the experiments, the objects were counter-balanced, in terms of their physical complexity and emotional neutrality. A preference index, that is, the ratio of time spent exploring either of the two objects (training session) or the novel object (retention test session) over the total time spent exploring both objects, was used.

### Gene expression analysis by quantitative real-time PCR

*Nrf2 (Nfe2l2)* and *Keap1* mRNA: At juvenile (P28) stage, mice were sacrificed, and their brains were removed for quantification of gene expression of *Nrf2 (Nfe2l2)* and *Keap1*. The frontal cortex and hippocampus were quickly dissected on ice from whole brain. A quantitative RT-PCR system (Step One Plus, Thermo Fisher Scientific, Yokohama, Japan) was used to measure mRNAs. The specific mRNA transcripts were quantified by TaqMan Gene Expression assays (Thermo Fisher Scientific, Yokohama, Japan). Expression levels of *Nfe2l2* (Mm00477784_m1), and *Keap1* (Mm00497268_m1) were measured. Total RNA was extracted by use of an RNeasy Mini Kit (Qiagen, Hilden, Germany). The purity of total RNA was assessed by Bio photometer plus (Eppendorf, Hamburg, Germany). The RNA samples were used in the first strand cDNA synthesis with High Capacity cDNA Reverse Transcription Kit (#4368813 Thermo Fisher Scientific, Yokohama, Japan). All samples were tested in triplicate and average values were used for quantification. The average values were normalized to Vic-labeled *Actb* mRNA (#4352341E: pre-developed TaqMan Assay Reagents, Thermo Fisher Scientific, Yokohama, Japan).

*Sfi1* mRNA: The expression levels of mouse *Sfl1* (Mm03039570_m1) were measured. Total RNA was extracted by use of an RNeasy Mini Kit (Qiagen, Hilden, Germany). The purity of total RNA was assessed by Bio photometer plus (Eppendorf, Hamburg, Germany). The RNA samples were used in the first strand cDNA synthesis with High Capacity cDNA Reverse Transcription Kit (#4368813 Thermo Fisher Scientific, Yokohama, Japan). All samples were tested in triplicate and average values were used for quantification. The average values were normalized to Vic-labeled *Actb* mRNA (#4352341E: pre-developed TaqMan Assay Reagents, Thermo Fisher Scientific, Yokohama, Japan).

### Immunohistochemistry for Parvalbumin (PV)

Immunohistochemistry of PV was performed as reported previously^28^. Mice were anesthetized with 5% isoflurane and sodium pentobarbital (50 mg/kg), and perfused transcardially with 10 mL of physiological saline, followed by 40 mL of ice-cold 4% paraformaldehyde in 0.1 M phosphate buffer (pH 7.4). Brains were removed from the skulls and post fixed overnight at 4 °C in the same fixative. For the immunohistochemical analysis, 50 μm-thick serial, coronal sections of brain tissue were cut in ice-cold 0.01 M phosphate buffered saline (pH 7.5) using a vibrating blade microtome (VT1000s, Leica Microsystems AG, Wetzlar, Germany). Free-floating sections were treated with 0.3% H_2_O_2_ in 50 mM Tris-HCl saline (TBS) for 30 min and ware blocked in TBS containing 0.2% Triton X-100 (TBST) and 1.5% normal serum for 1 h at room temperature. The samples are then incubated for 24 h at 4 °C with rabbit polyclonal anti-parvalbumin (PV) antibody (1:5,000, Swant, Bellin zona, Switzerland). The sections were washed three times in TBS and then processed using the avidin-biotin-peroxidase method (Vectastain Elite ABC, Vector Laboratories, Inc., Burlingame, CA, USA). Sections ware incubated for 3 min in a solution of 0.25 mg/mL DAB containing 0.01% H_2_O_2_. Then, sections were mounted on gelatinized slides, dehydrated, cleared, and cover slipped under Permount^®^ (Fisher Scientific, Fair Lawn, NJ, USA). The sections were imaged, and the staining intensity of PV immunoreactivity in the inflalimbic (IL) and prelimbic (PrL) regions of mPFC was analyzed using a light micro-scope equipped with a CCD camera (Olymups IX70, Tokyo, Japan) and the SCION IMAGE software package. Images of sections within mPFC region were captured using a 100× objective with a Keyence BZ-X700 microscope (Keyence Corporation, Osaka, Japan).

### RNA-sequencing and gene ontology analysis

Total RNA from prefrontal cortex (PFC) and hippocampus were isolated using the RNeasy Mini Kit (Qiagen) according to the manufacturer’s instructions. After RNA quality check (average of RIN values = 8) with RNA nano kit (Agilent Technologies) on Agilent Bioanalyzer (Agilent Technologies), 4 μg of pooled total RNA (5 samples in each group) were used for preparing cDNA libraries with the TruSeq RNA Sample Preparation Kit v2 (Illumina, San Diego, CA, USA), following the manufacturer’s instructions. The derived cDNA libraries were analyzed on an Agilent Bioanalyzer with DNA 1000 kit and quantified by qPCR using KAPA Library Quantification Kit (KAPA Bio systems, Wilmington, MA, USA). Twelve libraries with a unique barcode were pooled in three lanes and clusters were generated on a cBot (Illumina) to obtain 100 bp single reads in a HiSeq. 2500 sequencer (Illumina). Generation of demultiplexed fastq files was performed using bcl2fastq ver. 2.17 (Illumina) and 414 million sequence reads were obtained in total. Filtering, mapping, and differential expression analysis were performed using CLC Genomics Workbench software ver. 8.5 (Qiagen). The raw sequence reads were filtered to exclude adapter sequences, ambiguous nucleotides, and low-quality sequences and the retained sequences were aligned against the mouse genome (mm10). Analysis of the biological functions was performed using Ingenuity Pathway Analysis software (IPA, Ingenuity, Redwood City, CA, USA)(http://www.ingenuity.com) and gene set enrichment analysis (GSEA)(http://software.broadinstitute.org/gsea/index.jsp). RNA sequencing data have been deposited to the DDBJ Sequence Read Archive (DRA) and are available at the accession number DRA006055.

### Western blot of SFI1 protein in the postmortem brain samples from schizophrenia

Postmortem brain samples (n = 15) from schizophrenia and controls (n = 15)(Table 1) were obtained from the Neuropathology Consortium of the Stanley Medical Research Institute (MD, USA)^54,55^. Basically, the tissue samples were homogenized in Laemmli lysis buffer. Aliquots (50 μg for human samples) of protein were measured using a DC protein assay kit (Bio-Rad, Hercules, CA) and incubated for 5 min at 95 °C with an equal volume of a mixture of 125 mM Tris/HCl, pH 6.8, 20% glycerol, 0.1% bromophenol blue, 10% β-mercaptoethanol, and 4% sodium dodecyl sulfate, then and subjected to sodium dodecyl sulfate-polyacrylamide gel electrophoresis, using 10% mini-gels (Mini-PROTEAN® TGX™ Precast Gel; Bio-Rad, CA, USA). Proteins were transferred onto polyvinylidene difluoride membranes using a Trans Blot Mini Cell (Bio-Rad). For immunodetection, the blots were blocked with 2% bovine serum albumin in TBST (Tris-buffered saline + 0.1% Tween-20) for 1 h at room temperature (RT), and incubated overnight at 4 °C with primary antibodies. The following primary antibodies were used: SFI1 (Cat#: 13550–1-AP, 1: 1,000, Proteintech Japan, Tokyo, Japan). The next day, the blots were washed three times with TBST and incubated with horseradish peroxidase conjugated anti-rabbit antibody (1:5000) for 1 hour at RT. After three final washes with TBST, the bands were detected using enhanced chemiluminescence plus the Western Blotting Detection system (GE Healthcare Bioscience). The blots then were washed three times with TBST and incubated with a primary antibody against β-actin. Images were captured with a Fuji LAS3000-mini imaging system (Fujifilm, Tokyo, Japan) and immunoreactive bands were quantified.

### RT-qPCR of *SFI1* mRNA in the hair follicle samples from patients with schizophrenia

All samples (Table 2) of scalp hair-follicle were used as described in the previous work^43,53^. As described, expression levels of *SFI1* gene (Hs01564965_m1) were measured. Total RNA was extracted by use of an RNAqueous-Micro kit (Ambion, Grand Island, New York). The RNA samples were used in the first strand cDNA synthesis with SuperScript VILO Master Mix (#11755050 Thermo Fisher Scientific, Yokohama, Japan). All samples were tested in triplicate and average values were used for quantification based on the standard curve method. The average values were normalized to *GAPDH* mRNA (Hs02758991_g1: VIC-labeled TaqMan Gene Expression Assays, Thermo Fisher Scientific, Yokohama, Japan).

### Statistical analysis

All data are shown as mean ± standard error of the mean (S.E.M.) for animal data and mean ± standard deviation (S.D.) for human data. The data of locomotion, NORT, PV-immunostaining at juvenile stage, postmortem human brain and hair-follicle samples were analyzed by Student’s t-test. The data of locomotion, NORT, and PV-immunostaining at adult stage were analyzed by two-way analysis of variance (ANOVA), followed *post-hoc* Bonferroni test. Significance for results was set at P < 0.05. Significance for the GSEA was set at the false discovery rate (FDR) q < 0.25.
